# Oral nano-antioxidants improve sleep by restoring intestinal barrier integrity and preventing systemic inflammation

**DOI:** 10.1093/nsr/nwad309

**Published:** 2023-12-04

**Authors:** Zhanfeng Wu, Lei Liu, Lei Li, Xinran Cao, Wang Jia, Xiaodan Liao, Zhongpu Zhao, Hedong Qi, Guoqiang Fan, Huiqiang Lu, Chunying Shu, Mingming Zhen, Chunru Wang, Chunli Bai

**Affiliations:** Beijing National Laboratory for Molecular Sciences, Key Laboratory of Molecular Nanostructure and Nanotechnology, CAS Research/Education Center for Excellence in Molecular Sciences, Institute of Chemistry, Chinese Academy of Sciences, Beijing 100190, China; University of Chinese Academy of Sciences, Beijing 100049, China; Beijing National Laboratory for Molecular Sciences, Key Laboratory of Molecular Nanostructure and Nanotechnology, CAS Research/Education Center for Excellence in Molecular Sciences, Institute of Chemistry, Chinese Academy of Sciences, Beijing 100190, China; University of Chinese Academy of Sciences, Beijing 100049, China; Beijing National Laboratory for Molecular Sciences, Key Laboratory of Molecular Nanostructure and Nanotechnology, CAS Research/Education Center for Excellence in Molecular Sciences, Institute of Chemistry, Chinese Academy of Sciences, Beijing 100190, China; University of Chinese Academy of Sciences, Beijing 100049, China; Beijing National Laboratory for Molecular Sciences, Key Laboratory of Molecular Nanostructure and Nanotechnology, CAS Research/Education Center for Excellence in Molecular Sciences, Institute of Chemistry, Chinese Academy of Sciences, Beijing 100190, China; University of Chinese Academy of Sciences, Beijing 100049, China; Beijing National Laboratory for Molecular Sciences, Key Laboratory of Molecular Nanostructure and Nanotechnology, CAS Research/Education Center for Excellence in Molecular Sciences, Institute of Chemistry, Chinese Academy of Sciences, Beijing 100190, China; University of Chinese Academy of Sciences, Beijing 100049, China; Beijing National Laboratory for Molecular Sciences, Key Laboratory of Molecular Nanostructure and Nanotechnology, CAS Research/Education Center for Excellence in Molecular Sciences, Institute of Chemistry, Chinese Academy of Sciences, Beijing 100190, China; University of Chinese Academy of Sciences, Beijing 100049, China; Beijing National Laboratory for Molecular Sciences, Key Laboratory of Molecular Nanostructure and Nanotechnology, CAS Research/Education Center for Excellence in Molecular Sciences, Institute of Chemistry, Chinese Academy of Sciences, Beijing 100190, China; University of Chinese Academy of Sciences, Beijing 100049, China; Beijing National Laboratory for Molecular Sciences, Key Laboratory of Molecular Nanostructure and Nanotechnology, CAS Research/Education Center for Excellence in Molecular Sciences, Institute of Chemistry, Chinese Academy of Sciences, Beijing 100190, China; University of Chinese Academy of Sciences, Beijing 100049, China; School of Pharmacy, Wenzhou Medical University, Wenzhou 325000, China; Center for Drug Screening and Research, School of Geography and Environmental Engineering, Gannan Normal University, Ganzhou 341000, China; Beijing National Laboratory for Molecular Sciences, Key Laboratory of Molecular Nanostructure and Nanotechnology, CAS Research/Education Center for Excellence in Molecular Sciences, Institute of Chemistry, Chinese Academy of Sciences, Beijing 100190, China; University of Chinese Academy of Sciences, Beijing 100049, China; Beijing National Laboratory for Molecular Sciences, Key Laboratory of Molecular Nanostructure and Nanotechnology, CAS Research/Education Center for Excellence in Molecular Sciences, Institute of Chemistry, Chinese Academy of Sciences, Beijing 100190, China; University of Chinese Academy of Sciences, Beijing 100049, China; Beijing National Laboratory for Molecular Sciences, Key Laboratory of Molecular Nanostructure and Nanotechnology, CAS Research/Education Center for Excellence in Molecular Sciences, Institute of Chemistry, Chinese Academy of Sciences, Beijing 100190, China; University of Chinese Academy of Sciences, Beijing 100049, China; Beijing National Laboratory for Molecular Sciences, Key Laboratory of Molecular Nanostructure and Nanotechnology, CAS Research/Education Center for Excellence in Molecular Sciences, Institute of Chemistry, Chinese Academy of Sciences, Beijing 100190, China; University of Chinese Academy of Sciences, Beijing 100049, China

**Keywords:** sleep deprivation, fullerene nano-antioxidants, brain-gut axis, reactive oxygen species, inflammation

## Abstract

Sleep deprivation (SD) is a severe public health threat that can cause systemic inflammation and nerve damage. Few effective and side-effect-free drugs are available to address SD. However, the bidirectional communications between the brain and gut provide new strategies for anti-SD therapeutics. Here we explored oral delivery of fullerene nano-antioxidants (FNAO) in the SD model to improve sleep by regulating abnormal intestinal barrier and systemic inflammation via the brain-gut axis. SD caused excessive reactive oxygen species (ROS) production and hyperactive inflammatory responses in the intestines of zebrafish and mouse models, leading to disturbed sleep patterns and reduced brain nerve activity. Of note, based on the property of the conjugated *π* bond of the C_60_ structure to absorb unpaired electrons, oral FNAO efficiently reduced the excessive ROS in the intestines, maintained redox homeostasis and intestinal barrier integrity, and ameliorated intestinal and systemic inflammation, resulting in superior sleep improvement. Our findings suggest that maintaining intestinal homeostasis may be a promising avenue for SD-related nerve injury therapy.

## INTRODUCTION

As a pervasive and prominent occurrence in modern society, sleep deprivation (SD) is regarded as a public health epidemic with reduced sleep or decreased sleep quality due to stress, anxiety and depression [1]. The main feature of SD is the reduction of sleep time, and numerous studies have indicated that severe SD could lead to immunologic dysfunction [2], cardiovascular diseases [3], neurodegenerative diseases [4], diabetes [5] and other diseases, even increasing the risk of death [6]. However, there are few effective and side-effect-free drugs that focus on SD due to its complex pathological features. Researchers have explored many strategies for treatment of SD. For example, sleep disturbances or disorders, including SD, can affect the secretion of neurotransmitters (melatonin, 5-HT, dopamine, gamma-aminobutyric acid, etc.) [7–9]. Researchers use melatonin receptor agonists to regulate circadian rhythm disorders [10]. Meanwhile, selective 5-HT reuptake inhibitors targeting 5-HT receptors can increase 5-HT concentrations in the synaptic gap to improve depression-related sleep disorders [11]. While melatonin receptor agonists and selective serotonin reuptake inhibitors can regulate sleep homeostasis to some extent [12], they only target specific molecules and can only temporarily restore disordered neurotransmitters to normal levels. Thus, they cannot fundamentally improve sleep or reverse the systemic damage caused by SD. Also, small-molecule drugs often require higher doses or longer sustained courses of treatment to exert their critical effects, and the side-effects they bring to the body cannot be ignored. Coupled with drug dependence and safety concerns, there is no reliable drug to prevent or treat diseases caused by SD. Hence, it is of pressing importance to explore novel mechanisms and strategies to mitigate the morbidity associated with SD.

Recent research has identified that sleep communicates closely with the gut [13]. As a crucial peripheral immune system, the gut contains 70% of the immune lymphocyte cells in the human body. In particular, the intestinal epithelial barrier hinders the damage of external risk factors to the body, playing a critical role in regulating systemic immunity homeostasis [14]. Additionally, the studies on the brain-gut axis further elucidate the bidirectional communications between the gut and brain [15–17]. Once sleep deprived, it could be a risk factor for more frequent episodes of inflammatory bowel disease, which in turn worsens sleep abnormalities [18]. The latest studies have revealed that SD leads to the accumulation of excessive reactive oxygen species (ROS), specifically in the intestine [19]. These could result in high expressions of pro-inflammatory factors and continuous releases of danger signals into the blood, which leads to systemic inflammation and injury to nerves [20,21]. Therefore, in the intestine, researchers have used oral antioxidants, or modified antioxidant enzyme genes, to targeted peroxides to reduce the intestinal damage caused by SD in animal models [19]. However, there are few studies investigating the effects of antioxidants on sleep quality or sleep duration.

Fullerene, as a representative of carbon nanomaterials, has a wide range of applications in the biomedical field. Previous studies have reported that fullerene nanoparticles have superior antioxidant activity and anti-inflammatory properties [22–26]. Due to the large conjugated *π* bonds with high electron affinity on its carbon cage, it can efficiently capture the unpaired electrons of excessive free radicals at the site of lesions and regulate the body's redox balance [27]. Emerging studies showed that fullerene nanoparticles can restore damaged intestinal barrier function and further hinder entry of harmful lipopolysaccharides (LPSs) into the circulation of atherosclerotic mice [28]. Importantly, SD is closely related to the occurrence and development of cardiovascular diseases such as atherosclerosis [29]. This encourages us to study the effects of fullerene nanomaterials on both SD and sleep quality. Additionally, oral administration is a more commonly used method for administering small-molecule drugs and traditional medicines, as it offers better patient compliance compared to injection [30]. Given the central role of intestinal ROS in severe SD [13] and the efficient ability of fullerenes to eliminate ROS [22,24], we attempted to explore the role of oral administration of fullerene nanoparticles as an antioxidant in SD research.

**Figure 1.**
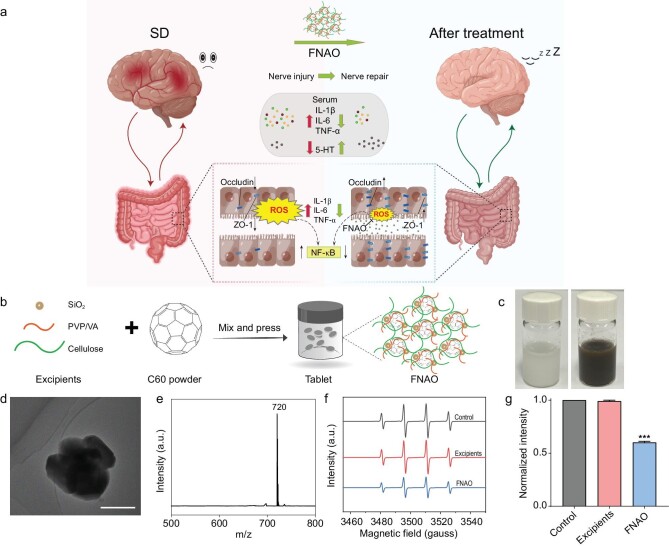
Schematic depiction and characterizations of FNAO. (a) SD improvement and anti-nerve injury mechanism diagram of FNAO. (b) Schematic diagram of the synthesis of FNAO. (c) Representative pictures of FNAO dispersion liquid (the left picture shows the dispersion state of pharmaceutical excipients without C_60_, and the right picture shows the dispersion state of FNAO). (d) TEM image of FNAO. Scale bar: 500 nm. (e) The MALDI-TOF-MS of FNAO. (f) The ESR spectrum of the hydroxyl radicals captured by DMPO after treatment with FNAO (blue line) and pharmaceutical excipients without C_60_ (red line), using water as a blank control (blank line). a.u., arbitrary unit. (g) The quantitative statistics intensity of ESR. Mean and SEM; *n* = 3 independent experiments. Differences were assessed by ANOVA and symbolized as follows: ***P < 0.001 compared with the control group.

In this work, we report for the first time that orally delivered fullerene nano-antioxidants (FNAO) are adopted to restore intestinal barrier integrity and systematically regulate the inflammatory microenvironment by acting on the brain-gut axis to achieve superior improvement in SD (Fig. 1a). FNAO could reduce excessive intestinal ROS and improve intestinal barrier function both in zebrafish and mouse models, thereby restraining intestinal and systemic inflammatory responses. More importantly, FNAO remarkably avoids SD-induced neuroinflammation damage, conserving circadian regulation and sleep homeostasis. Of note, following oral administration, FNAO is merely distributed in the intestine without diffusion into the whole body and could be excreted without causing apparent toxicity to the major organs. Our work provides a powerful candidate for improving SD and broadens the mind for therapy of SD-related nerve injury by maintaining intestinal homeostasis.

## RESULTS AND DISCUSSION

### Preparation and characterization of FNAO

First, we prepared FNAO with an effective free radical scavenging capacity through a simple method. The FNAO were prepared by pure C_60_ powder mixing with different types of frequently-used medical excipients for solubilization and disintegration, including microcrystalline cellulose, carboxymethyl cellulose, copovidone (PVP/VA) and silicon dioxide (SiO_2_) (Fig. 1b). To be orally delivered, they were compressed into tablets and dissolved in ultrapure water to form a dispersion system for further use (Fig. 1c). Subsequently, transmission electron microscopy (TEM) was adopted to determine the morphology and size of FNAO, which indicated that the particle size was around 1 *μ*m (Fig. 1d). Matrix-assisted laser desorption ionization-time of flight mass spectrometry (MALDI-TOF-MS) verified the presence of m/z 720 of C_60_ in the FNAO without the other peaks (Fig. 1e). Further, the capacity of FNAO to eliminate hydroxyl radicals (•OH) *in vitro* was determined by electron spin resonance (ESR). The •OH were generated from H_2_O_2_ in the presence of ultraviolet (UV) light and captured by 5,5-dimethyl-1-pyrroline N-oxide (DMPO). Compared with the pharmaceutical excipients group without C_60_, which did not have any ability to scavenge ROS, the FNAO had a significant scavenging efficiency on •OH (Fig. 1f and g).

### FNAO reduced oxidative stress and inflammatory response caused by SD in the zebrafish gut

**Figure 2.**
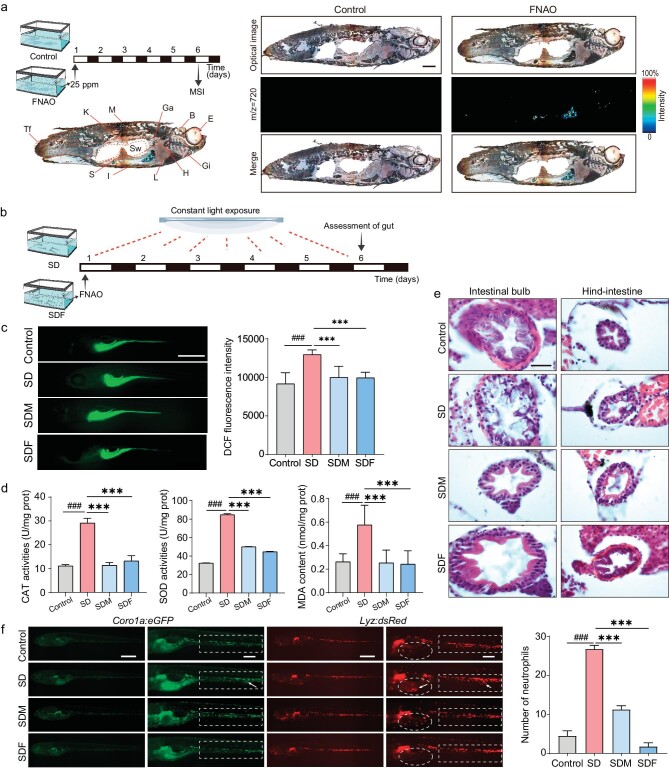
Assessments of FNAO on the levels of ROS and inflammatory-associated immune cells in the gut of zebrafish. (a) The optical images of zebrafish tissues and FNAO-related MALDI signal ([M-H]- at m/z 720) under 5 days’ exposure time. E = Eye; B = Brain; Gi = Gill; H = Heart; L = Liver; Ga = Gallbladder; Sw = Swim bladder; I = Intestine; M = Muscle; K = Kidney; S = Spleen; Tf = Tail fin. (b) Schematic diagram of SD improvement study by FNAO in zebrafish. (c) The DCF fluorescence images (left) and quantitative statistics intensity (right) in gut section of 5 days sleep-deprived zebrafish treated by FNAO. Scale bar, 500 *μ*m. Mean and SEM; *n* = 3 independent experiments. Differences were assessed by ANOVA and symbolized as follows: ^###^*P* < 0.001 compared with the control group, ***P < 0.001 compared with the SD group. (d) Measurements of the level of CAT activities, SOD activities, and MDA content of the zebrafish after 5 days of SD. (e) Images of H&E staining slices of intestinal bulb and hind-intestine in 5 days sleep-deprived zebrafish after FNAO treatment. Scale bar, 25 *μ*m. (f) The fluorescence images of neutrophils and macrophages in the gut of 5 days sleep-deprived zebrafish. Scale bars, 500 *μ*m (left), 200 *μ*m (right). The quantitative statistics of the number of neutrophils (right) in elliptic dashed space; *n* = 3 independent experiments. Differences were assessed by ANOVA and symbolized as follows: ^###^*P* < 0.001 compared with the control group, ***P < 0.001 compared with the SD group.

Zebrafish, as a new model organism, show great advantages in SD-related research. Compared with rats and mice, zebrafish could better simulate mammalian sleep structure as it moves during the day and sleeps at night [31]. Before the formal SD experiment, an *in-situ* matrix-assisted laser desorption ionization mass spectrometry imaging (MALDI-MSI) detection method was applied to investigate the visualization of tissue distributions of FNAO in zebrafish. Figure 2a displays the optical images of adult zebrafish and the selected ion images of C_60_ ([M-H]-at m/z 720.0) in various tissues after a 5-day exposure period. Compared with the control group without FNAO, the C_60_ of the FNAO group was mainly distributed in the intestine (especially in the intestinal contents), with very little in the gills (inevitably brought in by respiration). It indicated that fullerene (C_60_) as the active ingredient of FNAO suspension could directly act on the intestine of zebrafish rather than other tissues, and that the properties of C_60_ are stable in the gastrointestinal tract, which is highly consistent with our previous biodistribution study of FNAO [28].

Since persistent SD can lead to ROS accumulation and damage caused by systemic oxidative stress [19] especially in the gut [13], we first examined the effects of FNAO on the gut of SD zebrafish. We first established a SD model in zebrafish larvae by continuous illumination. Briefly, 6-day-old zebrafish larvae were exposed to 160 *lux* (unit of light illuminance) white light for 5 days, thus the zebrafish larvae were deprived of sleep (Fig. 2b). Due to the organism's powerful balance mechanism, there may be varying degrees of repair of SD damage after the cessation of SD [32]. Therefore, we chose to administer FNAO during continuous SD to reflect the role of FNAO in SD as much as possible, eliminating interference from self-repair mechanisms in the organism. We treated the SD zebrafish larvae with daily FNAO suspensions for 5 days with 2 *μ*g/mL/day (SDF group), using the melatonin (1 *μ*mol/L/day) as a positive control (SDM group). Subsequently, the ROS were examined after the treatment by FNAO and melatonin using a ROS sensitive probe 2′,7′-Dichlorofluorescein diacetate (DCFH-DA) in the SD larvae. It showed that excessive ROS were particularly generated in the gut by SD (Fig. 2c). Notably, FNAO suspensions reduced the DCF fluorescence intensity in the SDF group. Importantly, the DCF fluorescence intensity was much lower in the SDF group than that in the SDM group. Furthermore, we examined the effects of FNAO on overall oxidative stress in sleep-deprived zebrafish. Thirty AB zebrafish were taken from each group, ground on ice, and total protein was extracted and measured for concentration. The expression of the target molecules was detected according to the manufacturer's instructions. After 1 day of SD, the overall levels of catalase (CAT), superoxide dismutase (SOD), and malonaldehyde (MDA) in zebrafish showed little variation among the groups (Supplementary Fig. S1). After 5 days of SD, the levels of CAT, SOD, and MDA tended to increase. Of note, they were decreased almost back to the control group level after FNAO and melatonin treatment (Fig. 2d). This indicated that the oxidative stress levels in zebrafish increased with prolonged SD, and FNAO could actively regulate redox balance during the process of SD. The overproduction of ROS and oxidative stress would further lead to the occurrence of inflammation, and the cell structure would be vulnerable to damage. Thus, we used hematoxylin-eosin (H&E) staining to evaluate the pathology structure of the gut in zebrafish larvae, especially the intestinal bulb and hind-intestine (Fig. 2e). Our results revealed that the intestinal cavities had regular shape, the intestinal villus owned complete structure and the outline of intestinal epithelial cells were clear in the non-SD group (control group). On the contrary, the gut was severely damaged in the SD group. Specifically, the intestinal villus was exfoliated and intestinal epithelial cells were severely disrupted after SD. Interestingly, the villus and epithelial structures of the intestine were observably repaired after FNAO treatment. It indicated that FNAO could inhibit inflammation and improve the intestinal environment. To clarify the mechanism by which FNAO eliminates intestinal inflammation, we selected *Tg(lyz: DsRed2; coro1a: EGFP)* transgenic larvae as the optimal tool, which selectively expressed red fluorescent protein to track the neutrophils and green fluorescent protein to track the macrophages [33–35]. We established the SD model in transgenic larvae and treated them with FNAO using the same method. The results showed that both the macrophages and neutrophils were remarkably aggregated in the gut of SD larvae, especially in the intestinal bulb (labeled with ellipsoid) and hind-intestine (Fig. 2f). Of note, the aggregations of macrophages and neutrophils had dramatically vanished after FNAO suspension treatment. This indicated that FNAO improved intestinal damage by regulating the aggregation and migration of zebrafish macrophages and neutrophils. Taken together, FNAO significantly reduced oxidative stress and inflammation caused by SD in the zebrafish gut.

### FNAO improved sleep in sleep-deprived zebrafish

**Figure 3.**
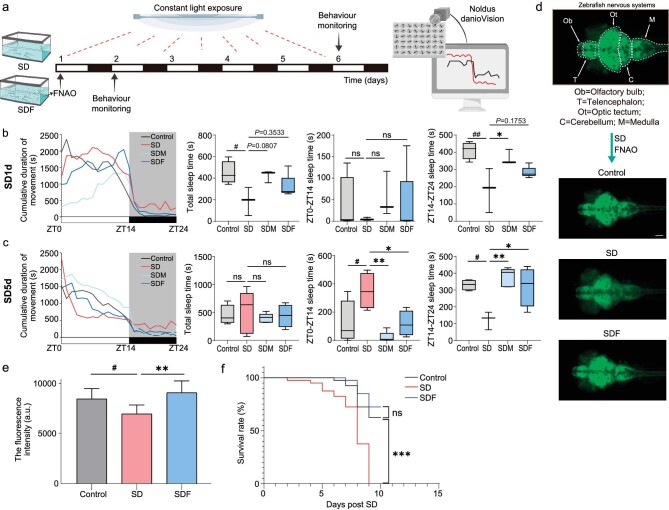
Sleep improvement effects of FNAO on SD in zebrafish. (a) Schematic diagram of SD improvement study in zebrafish. (b) Sleep behavior and sleep time of zebrafish on day 2 (after 1 day of SD) as reflected by the cumulative duration of movement; *n* = 5 independent experiments. (c) Sleep behavior and sleep time of zebrafish on day 6 (after 5 days of SD) as reflected by the cumulative duration of movement; *n* = 5 independent experiments. (d) The increased green fluorescence intensity of neurons in the sleep-deprived zebrafish brain after FNAO treatment. Scale bars, 100 *μ*m. (e) Quantification. (f) Overall Kaplan–Meier survival curves of sleep-deprived (red), FNAO therapy (blue) and non-deprived (black) zebrafish. Mean and SEM; *n* = 3 independent experiments. Differences were assessed by ANOVA and symbolized as follows: ns is no significant difference. ^#^*P* < 0.05, ^##^*P* < 0.01, ^###^*P* < 0.001 compared with the control group, *P < 0.05, **P < 0.01, ***P < 0.001 compared with the sleep-deprived group (SD).

Few studies have directly shown that improving the intestinal environment can improve sleep or reduce neuroinflammation [36,37], which greatly stimulated our exploration of the effects of FNAO on sleep and neurological function, in order to further confirm whether FNAO acts in the afferent direction (gut-to-brain) of the brain. Having shown the favorable effects of FNAO on the gut of sleep-deprived zebrafish, we sought to assess the impact of FNAO on sleep improvement in this model. The behavioral repertoire of zebrafish directly reflects their rest/awake states [38], so we used a behavior monitoring system to analyze the 24-h rest (sleep) time of zebrafish. First, zebrafish larvae were deprived of sleep for either 1 day or 5 days, and sleep-deprived larvae were treated with FNAO and melatonin, respectively. At zeitgeber time (ZT) 0 on days 2 and 6, we consecutively monitored the 24-h behavior changes of larvae under normal day-night environment (light for 14 h and dark for 10 h) by an automatic video tracking system (Fig. 3a). Total sleep time, ZT0–ZT14 sleep time and ZT14–ZT24 sleep time were analyzed, respectively. As shown in Fig. 3b, after 1 day of SD, compared to the control group, the cumulative activity time of zebrafish in the SD group significantly increased during the ZT14–ZT24 period. However, the cumulative activity time curve of the FNAO treatment group approached that of the control group and was superior to the melatonin treatment group (especially during the ZT0–ZT14 period). As SD extended to 5 days, the cumulative activity time of zebrafish in the SD group also significantly decreased during the ZT0–ZT14 period. Surprisingly, both FNAO and melatonin were able to improve this detrimental impact (Fig. 3c). After further analysis of sleep time, we found that the sleep-deprived zebrafish showed shortened duration of movement and lengthened sleep time during ZT0–ZT14, while showing longer duration of movement and shorter sleep time during ZT14–ZT24 compared with the control group. Interestingly, the prolonged total sleep time in SD zebrafish was almost back to a normal level after FNAO and melatonin treatments (Fig. 3b and c). Subsequently, we evaluated the potential effects of FNAO on brain nerves in sleep-deprived zebrafish using *Tg(elavl3: YC2)*, which labeled the neonatal neurons with YC2 that could be detected under fluorescence microscopy [39]. It showed a marked decline in fluorescence intensity after sleep loss in the SD group compared to the control group (Fig. 3d), which indicated it could be due to reduced transcription at *elavl*3 promoter and/or reduced translation of the reporter protein. Notably, the fluorescence intensity in neurons had increased back to almost normal levels after FNAO treatment (Fig. 3e). The results indicated that FNAO restored neuronal damage from SD-induction by increasing the transcription or translation of elavl3. We also recorded the survival rate of zebrafish larvae under SD. Each group consisted of 40 AB type zebrafish larvae, and the number of dead larvae in each group was recorded from the first day of SD until the day 10. The result indicated that continuous SD caused high mortality in zebrafish larvae, and after 8 days of SD, all zebrafish had died (Fig. 3f). Notably, the FNAO-treated group had a higher survival rate, suggesting that FNAO provided efficient protection to sleep-deprived zebrafish and prolonged their lifespan.

Taken together, for zebrafish larvae, FNAO treatment during SD could prevent pathological damage to the gut by regulating the body's redox balance and the migration of macrophages and neutrophils. In addition, SD was the cause of oxidative stress in organisms. As SD was prolonged from 1 day to 5 days, zebrafish behavior continued to change (Fig. 3b and c), and oxidative stress levels increased rapidly (Supplementary Figs S1 and 2d). Importantly, FNAO can efficiently regulate the body's redox balance to improve sleep in zebrafish. Furthermore, FNAO therapy could alleviate brain and nerve damage (Fig. 3d and e) in zebrafish larvae caused by SD, thereby extending their survival time (Fig. 3f). This indicated that even under conditions of continuous SD where the organism did not have enough time for self-repair, FNAO could effectively reduce the damage caused by prolonged SD and improve the organism's sleep condition. However, we still do not know how FNAO sent signals to the brain to improve sleep and reshape neural function. Due to the fact that C_60_ was exclusively distributed in the gut (Fig. 2a) and did not enter the bloodstream or brain tissue [28], we speculated that FNAO improved sleep by targeting the gut and regulating the balance of the intestinal environment.

### FNAO improved sleep in SD mice

**Figure 4.**
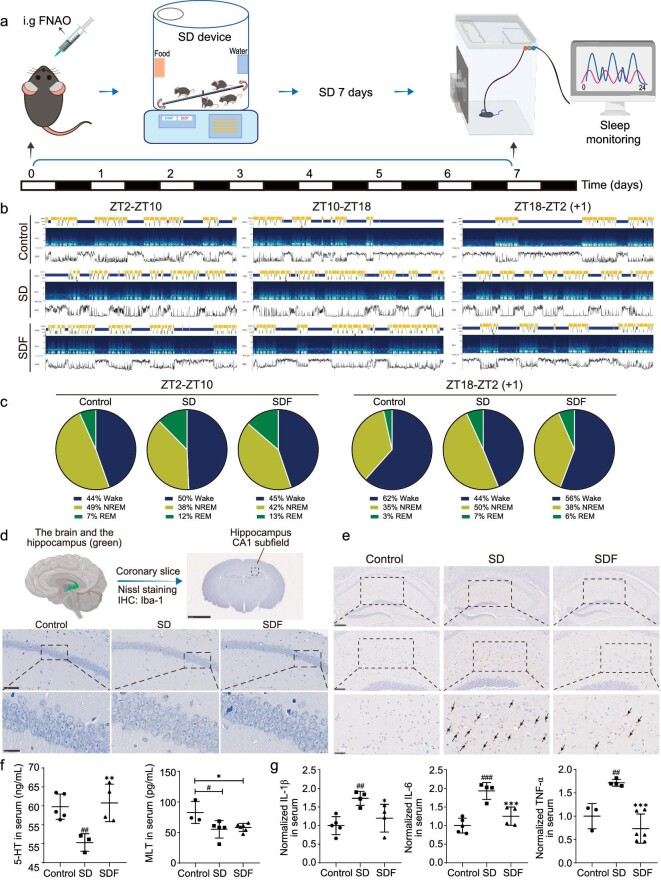
Effects of FNAO on SD in mice. (a) Schematic diagram of the mouse SD study. (b) EEG/EMG monitoring after 7 days of SD. (c) Quantified sleep structure by EEG/EMG (b). Rapid eye movement (REM) sleep, non-REM (NREM) sleep, and wakefulness (Wake) in non-deprived (non-SD or Control), sleep-deprived (SD) and FNAO treatment (SDF) C57BL/6J mice. (d) The Nissl staining in the hippocampus CA1 subfield (scale bar is 2.5 mm) and the corresponding magnification (scale bars: 100 *μ*m; 25 *μ*m). (e) The Iba-1 immunohistochemistry images in the hippocampus field (scale bar is 250 *μ*m) and the corresponding magnification (scale bars: 100 *μ*m; 25 *μ*m). (f) Measurements of the levels of 5-HT, Melatonin (MLT) and the normalized levels of IL-1*β*, IL-6, and TNF-*α* (g) in serum of the mice in the non-SD (Control), SD and FNAO treatment (SDF) groups via *ELISA* tests. Mean and SEM; *n* = 3 independent experiments. Differences were assessed by ANOVA and symbolized as follows: ^#^*P* < 0.05, ^##^*P* < 0.01, ^###^*P* < 0.001 compared with the control group (Control), *P < 0.05, **P < 0.01, ***P < 0.001 compared with the sleep-deprived group (SD).

Sleep regulation is conserved across species [40]. As zebrafish are small and cannot be easily dissected to study each organ in a molecular biology context from the brain to the gut, we chose C57BL/6J mice as the next research subjects to capture the signal exchange between the brain and gut in both the efferent (brain-to-gut) and afferent (gut-to-brain) directions [17]. First, a mouse model of SD was constructed. C57BL/6J mice were deprived of sleep for up to 7 days with gently continuous mechanical stimulation by a mouse rotating-stick SD meter. We treated SD mice with daily FNAO (80 mg/kg/day) and used the gold standard electroencephalogram (EEG)/electromyogram (EMG) monitoring [41] of mouse sleep to examine whether FNAO could improve sleep in SD mice (Fig. 4a). At ZT2 on day 8, we consecutively monitored the 24-h EEG/EMG of mice under normal day-night environment (light for 12 h and dark for 12 h) by the EEG/EMG monitoring system (Fig. 4b). We recorded the stages including Wake, NREM and REM in three periods: ZT2–ZT10, ZT10–ZT18 and ZT18–ZT2 (+1) (+1 was the next day). It showed that the sleep structures of mice after SD were obviously disrupted. Specifically, the sleep latency of the mice in the SD group became longer compared with those in the control group during ZT2–ZT10. Moreover, the sleep cycle was significantly fragmented in SD mice in the total 24-h record [the frequency of cycle replacement of awakening time (blue strip) and sleep time (yellow strip and green strip) was relatively quick]. Interestingly, these abnormalities in SD mice were observably improved after FNAO treatment. It could be found that the sleep latency of FNAO-treated mice was distinctly shortened, the phenomenon of sleep fragmentation was markedly reversed, and the sleep cycle mostly returned to normal. In particular, after FNAO treatment, the awakening time during ZT2–ZT10 was decreased from ∼50% to ∼45% in SD mice, and the total sleep time (the sum of REM sleep and NREM sleep) was increased to ∼55%. Also, the awakening time during ZT18–ZT2 (+1) was significantly increased from ∼44% to ∼56% in SD mice treated with FNAO, and the total sleep time was decreased to ∼44% after FNAO treatment (Fig. 4c). Together, FNAO notably improved the sleep status of SD mice.

After confirming the rescue of sleep fragmentation by FNAO in SD mice, we further studied the effect of FNAO treatment on brain nerve protection. First, neuron loss was one of the hallmarks in SD [42]. The Nissl body is one of the characteristic structures of neurons. To observe the effects on neuron loss by FNAO treatment, mouse brain tissues were fixed and paraffin-sealed. Then, we used the Nissl staining of the paraffin sections to evaluate the morphological changes in mice hippocampal neurons (Fig. 4d). We found that the Nissl bodies boundary of mice in the SD group had an unclear cell-boundary compared with the control group, and the nucleus of neurons were mostly karyolitic. Notably, the neurons had a clear nucleus, abundant Nissl bodies and regular sequence after FNAO treatment. These results suggested that FNAO maintained the normal morphological structure of Nissl bodies in SD mice. In addition, as important immune cells in the central nervous system (CNS), microglia actively regulate the immune function of the brain and reduce inflammation [43,44]. We assessed the contents of ionized calcium-binding adapter molecule 1 (Iba-1) by immunohistochemical staining, which is a marker of reactive microglia in the brain. It showed that the number of Iba-1 positive cells in the hippocampal CA1 region of the control group mice was less (Fig. 4e), indicating that microglia in the hippocampal region of mice were in a quiescent state. However, the number of Iba-1 positive cells in SD group mice was significantly increased, and the cell body became larger, accompanied by the deepened staining. In particular, compared with the SD group mice, Iba-1 positive cells decreased significantly in the FNAO treated group, suggesting that microglia returned to a relatively normal state. Collectively, these observations suggested that FNAO could protect mouse nerves from damage by inhibiting the excessive activation of CNS microglial cells.

We next assessed the effect of FNAO on neurotransmitters and systemic inflammation. The levels of representative neurotransmitters and inflammatory cytokines were assessed using an enzyme linked immunosorbent assay (*ELISA*). Serotonin, also referred to as 5-hydroxytryptamine (5-HT), is an important neurotransmitter that plays an important role in controlling sleep and arousal [45]. Besides, 5-HT could convert into melatonin (MLT), which is the dominant regulator of the sleep cycle and circadian rhythm [46]. As shown in Fig. 4f, the levels of 5-HT and melatonin in the serum of the SD group were much lower than those in the control group. After treatment with FNAO, the level of 5-HT returned to normal, but the melatonin level did not, which reminded us that after FNAO treatment, the upregulation rate of 5-HT seemed to be much higher than the conversion rate of 5-HT to melatonin. This suggested that FNAO may selectively regulate neurotransmitters through indirect means. In addition, FNAO improved sleep most likely through the 5-HT pathway rather than the melatonin pathway. Continuous SD can result in an increase in pro-inflammatory cytokines [47]. *ELISA* results showed that the levels of proinflammatory cytokine in serum, such as IL-1*β*, IL-6 and TNF-*α*, were increased after SD which were then significantly reduced after FNAO treatment (Fig. 4g).

The above results demonstrated that FNAO had a positive regulatory effect on sleep structure disorder in SD mice, which may be due to the significant regulatory effect of FNAO treatment on Nissl body dissolution and excessive activation of microglia in the CNS of SD mice. Furthermore, FNAO could capture the signal in the efferent direction of the brain, effectively reducing systemic inflammation and regulating the release of neurotransmitters in order to inhibit neural damage caused by SD stress.

### FNAO improved intestinal barrier function and inhibited intestinal inflammation by down-regulating the NF-*κ*B signaling pathway

**Figure 5.**
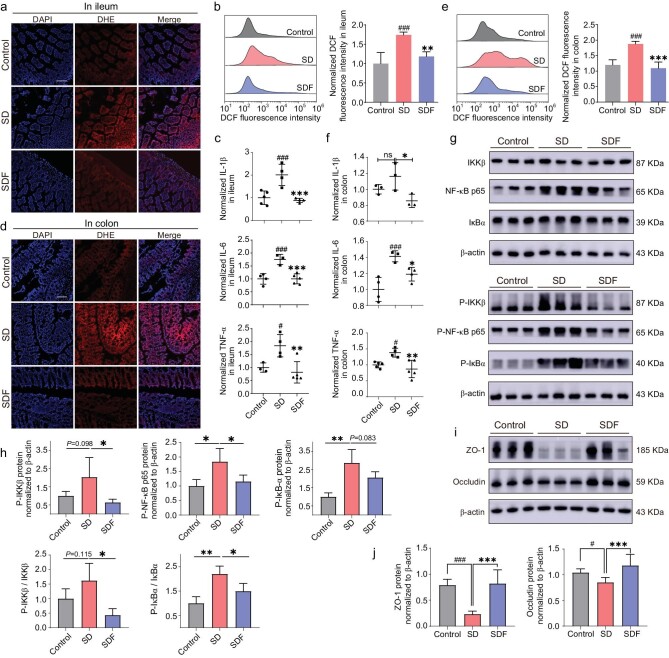
FNAO could improve intestinal barrier function and inhibit intestinal inflammation by reducing the activation of the NF-*κ*B signaling pathway. (a) Oxidized DHE reported high levels of reactive oxygen species (ROS) in the ileum (a) and the colon (d) of sleep-deprived mice (red) after 7 days. FNAO treatment reduced DHE fluorescence intensity. Scale bars, 100 *μ*m. The FCM analysis of the ROS levels in total ileum (b) and colon (e) cells of mice in non-deprived (non-SD or Control, gray), sleep-deprived (SD, pink) and FNAO treatment (SDF, purple) group, respectively. Quantification (right). FNAO significantly reduced the levels of inflammatory cytokines IL-1*β*, IL-6, and TNF-*α* caused by SD 7 days in the ileum (c) and colon (f). (g) Protein expression of IKK*β*, phosphorylated IKK*β*, NF-*κ*B p65, phosphorylated NF-*κ*B p65, I*κ*B*α*, and phosphorylated I*κ*B*α*. The quantification is shown in (h). (i) Protein expression of ZO-1 and Occludin in the small intestine after FNAO treatment. Quantification (Right, j). Mean and SEM; *n* = 3 independent experiments. Differences were assessed by ANOVA and symbolized as follows: ns is no significant difference. ^#^*P* < 0.05, ^##^*P* < 0.01, ^###^*P* < 0.001 compared with the control group (Control), *P < 0.05, **P < 0.01, ***P < 0.001 compared with the sleep-deprived group (SD).

After receiving signals of SD, the brain induced corresponding changes in the efferent direction (brain-to-gut) which can affect peripheral organs or tissues. In order to further explore whether the mechanism of FNAO improving sleep in mice was related to the intestine, we next detected the contents of ROS in both the ileum and colon of sleep-deprived mice using cryo-sectioning and flow cytometry. The superoxide anion level was detected by superoxide anion fluorescence probe dihydroethidium (DHE) staining based on the intestinal cryosection technique. The total intracellular ROS level was detected by DCFH-DA based on flow cytometry. For the ileum, as shown in Fig. 5a and b, FNAO could observably reduce the increased DHE as well as DCF fluorescence intensity in SD mice. In addition, we tested the levels of IL-1*β*, IL-6 and TNF-*α* in the ileum by *ELISA* kits. It revealed that the excessive ROS in the ileum mainly induced the high levels of these pro-inflammatory cytokines in SD mice, which were decreased after FNAO treatment (Fig. 5c). For the colon, the improvement trends on oxidative stress and inflammation by FNAO treatment were almost the same as those in the ileum (Fig. 5d–f). All the results indicated that FNAO significantly decreased the oxidative stress and inflammatory levels in the intestine of the sleep-deprived mice. To further explore the mechanism at molecular biological levels by which FNAO alleviated intestinal inflammation, we assessed protein levels along the NF-*κ*B signaling pathway in the ileum. In our work, western blot (WB) assays revealed that it activated the NF-*κ*B signaling pathway in the ileum after SD. We found that IKK*β*, NF-*κ*B p65 and I*κ*B-*α* showed almost no change after treatment with FNAO compared with the SD group (Supplementary Fig. S2). However, the phosphorylated IKK*β* (P-IKK*β*), phosphorylated NF-*κ*B p65 (P-NF-*κ*B p65) and phosphorylated I*κ*B-*α* (P-I*κ*B-*α*) were significantly decreased after FNAO treatment compared with the SD group (Fig. 5g and h). We demonstrated that oral FNAO could alleviate the inflammatory response in the intestines of sleep-deprived mice by inhibiting the excessive activation of the NF-*κ*B signaling pathway.

In addition, given that tight junction proteins play important roles in maintaining intestinal homeostasis, we further detected the levels of two critical tight junction proteins (ZO-1 and Occludin) in the ileum of SD mice using WB and immunofluorescent staining. As shown in Fig. 5i and j, the protein expression levels of both ZO-1 and Occludin were decreased in the ileum of the SD group compared with the control group. A similar conclusion was reached by immunofluorescence (Supplementary Fig. S3). It showed that ZO-1 protein in the ileal villous border was missing in the SD group. The deficiency of these permeability proteins may lead to damage of the intestinal barrier integrity, thus exposing the immune system to inflammatory stimuli from the intestinal environment, thereby creating a vicious cycle. Notably, compared with the SD group, FNAO significantly increased the protein expressions of the intestinal tight junction and repaired intestinal barrier function. These results indicated that FNAO was extremely important for maintaining homeostasis within the intestine and the systemic circulation of the organism, as studies have shown that metabolites derived from the intestine can enter the systemic circulation and even brain tissue through a compromised intestinal barrier, subtly influencing the host's sleep [48].

In conclusion, in sleep-deprived mice, the active ingredient C_60_ in orally administered FNAO materials could directly interact with the intestine, efficiently capturing unpaired electrons and significantly clearing excessive ROS (Fig. 1f). It also markedly reduced the levels of pro-inflammatory cytokines by regulating the NF-*κ*B signaling pathway. Furthermore, FNAO protects the systemic circulation and even brain tissues from SD-induced damage by increasing the expression of tight junction proteins of the intestine, which prevents inflammatory factors or other harmful substances from entering the systemic circulation. This suggested that in sleep-deprived mice, FNAO improved sleep by targeting the intestine, which may be the result of sending repair signals to the sleep-deprived brain via the brain-gut axis in the afferent direction (gut-to-brain). Although we are still unclear about how FNAO sends signals, and which other signaling molecules it sends (except for the neurotransmitter 5-HT), it is certain that the intestine is the target of FNAO. Furthermore, based on the ability of FNAO to directly eliminate excessive ROS in the intestine of mice (Fig. 5a, b, d and e), this may effectively intercept harmful stimuli at the source and prevent gut inflammation before compromising the integrity of the intestinal barrier function. Besides, we have demonstrated that oral C_60_ nano-antioxidants could relieve Parkinson's disease by regulation of the gut microbiota [49]. This suggests that FNAO may play a therapeutic role by regulating these microbiota. In addition to the excellent therapeutic effect, the safety of FNAO was reliable. The results of H&E staining indicated that FNAO had no significant toxicity (Supplementary Fig. S4) on major organs (heart, liver, spleen, lung, and kidney) after oral administration.

The brain and the gut play important roles in detecting, transmitting, and responding to internal and external environmental signals [50]. FNAO regulates host intestinal injury by directly modulating intestinal damage in the direction of gut-to-brain communication and transmit healthy signals to help restore homeostasis in the direction of brain-to-gut communication by modulating the release of neurotransmitters. Therefore, modulating the excessive ROS and intestinal barrier function in intestine tissues to improve systemic inflammatory response is a promising strategy for reversing SD and the nerve damage caused by SD. The study of the relationships between SD and the gut is in its infancy, and there are still many unknown communication relationships between the brain and gut. We should continue to study the mechanism of SD related to the intestinal environment and the relationship between intestinal injury and neuronal injury, which may expand the applications of nano-antioxidants in neurodegenerative diseases or other brain-gut axis disease.

## CONCLUSIONS

In summary, we develop a promising strategy by FNAO to remove intestinal excessive ROS and repair the intestine in order to improve sleep based on the close communications between brain and gut. One of the most notable findings in our study is that FNAO improves sleep mainly by reducing intestinal oxidative stress and systemic inflammatory response caused by SD in zebrafish and mouse models. We also observe that FNAO regulated the NF-*κ*B signaling pathway and repaired intestinal barrier function in SD mice. Furthermore, we certify that FNAO could be almost all excreted from the living body and cause no severe toxicity. This finding provides a powerful candidate for improving sleep and broadens the therapeutic approaches to SD-related nerve injury, which would be useful for studying the sleep mechanism of oral nanomaterials associated with the brain-gut axis.

## Supplementary Material

nwad309_Supplemental_File

## References

[1] McEwen BS . Sleep deprivation as a neurobiologic and physiologic stressor: allostasis and allostatic load. Metabolism 2006; 55: S20–3. 10.1016/j.metabol.2006.07.008 16979422

[2] Scheiermann C , Kunisaki Y, Frenette PS. Circadian control of the immune system. Nat Rev Immunol 2013; 13: 190–8. 10.1038/nri3386 23391992 PMC4090048

[3] Gangwisch JE , Heymsfield SB, Boden-Albala B et al. Short sleep duration as a risk factor for hypertension: analyses of the first National Health and Nutrition Examination Survey. Hypertension 2006; 47: 833–9. 10.1161/01.HYP.0000217362.34748.e0 16585410

[4] Wulff K , Gatti S, Wettstein JG et al. Sleep and circadian rhythm disruption in psychiatric and neurodegenerative disease. Nat Rev Neurosci 2010; 11: 589–99. 10.1038/nrn2868 20631712

[5] Cappuccio FP , D’Elia L, Strazzullo P et al. Quantity and quality of sleep and incidence of type 2 diabetes: a systematic review and meta-analysis. Diabetes Care 2010; 33: 414–20. 10.2337/dc09-1124 19910503 PMC2809295

[6] McAlpine CS , Kiss MG, Rattik S et al. Sleep modulates haematopoiesis and protects against atherosclerosis. Nature 2019; 566: 383–7. 10.1038/s41586-019-0948-2 30760925 PMC6442744

[7] Gerstner JR , Yin JCP. Circadian rhythms and memory formation. Nat Rev Neurosci 2010; 11: 577–88. 10.1038/nrn2881 20648063 PMC6544049

[8] Garritsen O , van Battum EY, Grossouw LM et al. Development, wiring and function of dopamine neuron subtypes. Nat Rev Neurosci 2023; 24: 134–52. 10.1038/s41583-022-00669-3 36653531

[9] Gassmann M , Bettler B. Regulation of neuronal GABAB receptor functions by subunit composition. Nat Rev Neurosci 2012; 13: 380–94. 10.1038/nrn3249 22595784

[10] Lewis S . Melatonin influence on circadian rhythms. Nat Rev Neurosci 2016; 17: 4.26656256

[11] Kantor S , Varga J, Kulkarni S et al. Chronic paroxetine treatment prevents the emergence of abnormal electroencephalogram oscillations in Huntington's Disease mice. Neurotherapeutics 2017; 14: 1120–33. 10.1007/s13311-017-0546-7 28653279 PMC5722757

[12] Morin CM , Benca R. Chronic insomnia. Lancet North Am Ed 2012; 379: 1129–41. 10.1016/S0140-6736(11)60750-2 22265700

[13] Vaccaro A , Kaplan Dor Y, Nambara K et al. Sleep loss can cause death through accumulation of reactive oxygen species in the gut. Cell 2020; 181: 1307–28. 10.1016/j.cell.2020.04.049 32502393

[14] Ma H , Tao W, Zhu S. T lymphocytes in the intestinal mucosa: defense and tolerance. Cell Mol Immunol 2019; 16: 216–24. 10.1038/s41423-019-0208-2 30787416 PMC6460495

[15] Fung TC , Olson CA, Hsiao EY. Interactions between the microbiota, immune and nervous systems in health and disease. Nat Neurosci 2017; 20: 145–55. 10.1038/nn.4476 28092661 PMC6960010

[16] Cryan JF , O’Riordan KJ, Cowan CSM et al. The microbiota-gut-brain axis. Physiol Rev 2019; 99: 1877–2013. 10.1152/physrev.00018.2018 31460832

[17] Agirman G , Yu KB, Hsiao EY. Signaling inflammation across the gut-brain axis. Science 2021; 374: 1087–92. 10.1126/science.abi6087 34822299

[18] Ananthakrishnan AN , Long MD, Martin CF et al. Sleep disturbance and risk of active disease in patients with Crohn's Disease and ulcerative Colitis. Clin Gastroenterol Hepatol 2013; 11: 965–71. 10.1016/j.cgh.2013.01.021 23376797 PMC3659204

[19] Gulec M , Ozkol H, Selvi Y et al. Oxidative stress in patients with primary insomnia. Prog Neuropsychopharmacol Biol Psychiatry 2012; 37: 247–51. 10.1016/j.pnpbp.2012.02.011 22401887

[20] Chennaoui M , Gomez-Merino D, Drogou C et al. Effects of exercise on brain and peripheral inflammatory biomarkers induced by total sleep deprivation in rats. J Inflamm 2015; 12: 56. 10.1186/s12950-015-0102-3 PMC4588685 26425116

[21] Bellesi M , de Vivo L, Chini M et al. Sleep loss promotes astrocytic phagocytosis and microglial activation in mouse cerebral cortex. J Neurosci 2017; 37: 5263–73. 10.1523/JNEUROSCI.3981-16.2017 28539349 PMC5456108

[22] Zhou C , Zhen M, Yu M et al. Gadofullerene inhibits the degradation of apolipoprotein B100 and boosts triglyceride transport for reversing hepatic steatosis. Sci Adv 2020; 6: eabc1586. 10.1126/sciadv.abc1586 32917715 PMC7556997

[23] Jia W , Zhen M, Li L et al. Gadofullerene nanoparticles for robust treatment of aplastic anemia induced by chemotherapy drugs. Theranostics 2020; 10: 6886–97. 10.7150/thno.46794 32550910 PMC7295067

[24] Li L , Zhen M, Wang H et al. Functional gadofullerene nanoparticles trigger robust cancer immunotherapy based on rebuilding an immunosuppressive tumor microenvironment. Nano Lett 2020; 20: 4487–96. 10.1021/acs.nanolett.0c01287 32407113

[25] Yu T , Zhen M, Li J et al. Anti-apoptosis effect of amino acid modified gadofullerene via a mitochondria mediated pathway. Dalton Trans 2019; 48: 7884–90. 10.1039/C9DT00800D 31080968

[26] Liao X , Zhao Z, Li H et al. Fullerene nanoparticles for the treatment of ulcerative colitis. Sci China Life Sci 2022; 65: 1146–56. 10.1007/s11427-021-2001-0 34735681

[27] Li X , Zhen M, Zhou C et al. Gadofullerene nanoparticles reverse dysfunctions of pancreas and improve hepatic insulin resistance for type 2 diabetes mellitus treatment. ACS Nano 2019; 13: 8597–608. 10.1021/acsnano.9b02050 31314991

[28] Su S , Zhen M, Zhou C et al. Efficiently inhibiting systemic inflammatory cascades by fullerenes for retarding HFD-fueled atherosclerosis. Adv Healthc Mater 2023; 12: 2202161. 10.1002/adhm.202202161 36623263

[29] McAlpine CS , Kiss MG, Rattik S et al. Sleep modulates haematopoiesis and protects against atherosclerosis. Nature 2019; 566: 383–7. 10.1038/s41586-019-0948-2 30760925 PMC6442744

[30] Durán-Lobato M , Niu ZG, Alonso MJ. Oral delivery of biologics for precision medicine. Adv Mater 2020; 32: e1901935. 31222910 10.1002/adma.201901935

[31] Oikonomou G , Prober DA. Attacking sleep from a new angle: contributions from zebrafish. Curr Opin Neurobiol 2017; 44: 80–8. 10.1016/j.conb.2017.03.009 28391131 PMC5659277

[32] Hendricks JC , Finn SM, Panckeri KA et al. Rest in Drosophila is a sleep-like state. Neuron 2000; 25: 129–38. 10.1016/S0896-6273(00)80877-6 10707978

[33] Hu Y , Jia K, Zhou Y et al. Rutin hydrate relieves neuroinflammation in zebrafish models: involvement of NF-κB pathway as a central network. Fish Shellfish Immunol 2023; 141: 109062. 10.1016/j.fsi.2023.109062 37678480

[34] Zhang R , Liu X, Li Y et al. Suppression of inflammation delays hair cell regeneration and functional recovery following lateral line damage in zebrafish larvae. Biomolecules 2020; 10: 1451. 10.3390/biom10101451 33081293 PMC7650643

[35] Cheng B , Zhang H, Hu JH et al. The immunotoxicity and neurobehavioral toxicity of zebrafish induced by famoxadone-cymoxanil. Chemosphere 2020; 247: 125870. 10.1016/j.chemosphere.2020.125870 31931321

[36] Wang Z , Chen W-H, Li S-X et al. Gut microbiota modulates the inflammatory response and cognitive impairment induced by sleep deprivation. Mol Psychiatry 2021; 26: 6277–92. 10.1038/s41380-021-01113-1 33963281

[37] Gao T , Wang Z, Dong Y et al. Role of melatonin in sleep deprivation-induced intestinal barrier dysfunction in mice. J Pineal Res 2019; 67: e12574. 10.1111/jpi.12574 30929267

[38] Rihel J , Prober DA, Arvanites A et al. Zebrafish behavioral profiling links drugs to biological targets and rest/wake regulation. Science 2010; 327: 348–51. 10.1126/science.1183090 20075256 PMC2830481

[39] Cheng B , Jiang F, Su ML et al. Effects of lincomycin hydrochloride on the neurotoxicity of zebrafish. Ecotoxicol Environ Saf 2020; 201: 110725. 10.1016/j.ecoenv.2020.110725 32474209

[40] Cirelli C . The genetic and molecular regulation of sleep: from fruit flies to humans. Nat Rev Neurosci 2009; 10: 549–60. 10.1038/nrn2683 19617891 PMC2767184

[41] Halassa MM , Florian C, Fellin T et al. Astrocytic modulation of sleep homeostasis and cognitive consequences of sleep loss. Neuron 2009; 61: 213–9. 10.1016/j.neuron.2008.11.024 19186164 PMC2673052

[42] Cao Y , Yang YB, Wu H et al. Stem-leaf saponins from Panax notoginseng counteract aberrant autophagy and apoptosis in hippocampal neurons of mice with cognitive impairment induced by sleep deprivation. J Ginseng Res 2020; 44: 442–52. 10.1016/j.jgr.2019.01.009 32372866 PMC7195596

[43] Giovannoni F , Quintana FJ. The role of astrocytes in CNS inflammation. Trends Immunol 2020; 41: 805–19. 10.1016/j.it.2020.07.007 32800705 PMC8284746

[44] Deczkowska A , Amit I, Schwartz M. Microglial immune checkpoint mechanisms. Nat Neurosci 2018; 21: 779–86. 10.1038/s41593-018-0145-x 29735982

[45] Monti JM . Serotonin control of sleep-wake behavior. Sleep Med Rev 2011; 15: 269–81. 10.1016/j.smrv.2010.11.003 21459634

[46] Halson SL . Sleep in elite athletes and nutritional interventions to enhance sleep. Sports Med 2014; 44: 13–23. 10.1007/s40279-014-0147-0 PMC4008810 24791913

[47] Mishra I , Pullum KB, Thayer DC et al. Chemical sympathectomy reduces peripheral inflammatory responses to acute and chronic sleep fragmentation. Am J Physiol Regul Integr Comp Physiol 2020; 318: R781–9. 10.1152/ajpregu.00358.2019 32130024 PMC7191417

[48] Needham BD , Funabashi M, Adame MD et al. A gut-derived metabolite alters brain activity and anxiety behaviour in mice. Nature 2022; 602: 647–53. 10.1038/s41586-022-04396-8 35165440 PMC9170029

[49] Li X , Deng R, Li J et al. Oral 60 fullerene reduces neuroinflammation to alleviate Parkinson's disease via regulating gut microbiome. Theranostics 2023; 13: 4936–51. 10.7150/thno.85711 37771782 PMC10526674

[50] Nicholson JK , Holmes E, Kinross J et al. Host-gut microbiota metabolic interactions. Science 2012; 336: 1262–7. 10.1126/science.1223813 22674330

