# Supplementary material for: Oral nano-antioxidants improve sleep by restoring intestinal barrier integrity and preventing systemic inflammation
Source: Natl Sci Rev. 2023 Dec 4;10(12):nwad309. doi: 10.1093/nsr/nwad309 (PMC10781441; doi:10.1093/nsr/nwad309)
Supplement: nwad309_Supplemental_File [file nwad309_supplemental_file.docx]

Supplementary Data

Oral Nano-antioxidants Improve Sleep by Restoring Intestinal Barrier Integrity and Preventing Systemic Inflammation

Zhanfeng Wu^1,2^, Lei liu^1,2^, Lei Li^1,2^, Xinran Cao^1,2^, Wang Jia^1,2^, Xiaodan Liao^1,2^, Zhongpu Zhao^1,2^, Hedong Qi^1,2^, Guoqiang Fan^3^, Huiqiang Lu^4^, Chunying Shu^1,2^, Mingming Zhen^1,2*^, Chunru Wang^1,2*^ and Chunli Bai^1,2*^

^1^Beijing National Laboratory for Molecular Sciences, Key Laboratory of Molecular Nanostructure and Nanotechnology, CAS Research/Education Center for Excellence in Molecular Sciences, Institute of Chemistry, Chinese Academy of Sciences, Beijing 100190, China

^2^University of Chinese Academy of Sciences, Beijing 100049, China

^3^School of Pharmacy, Wenzhou Medical University, Wenzhou 325000, China

^4^Center for Drug Screening and Research, School of Geography and Environmental Engineering, Gannan Normal University, Ganzhou 341000, China

**EXPERIMENTAL METHODS**

**Preparation and Characterization of FNAO*.***

Synthesis of FNAO referred to the previous research in our laboratory [1]. In a word, C_60_ solid powder, microcrystalline cellulose 0.2-1.2% (m/v), carboxymethyl cellulose 0.2-1.2% (m/v), copovidone 0.2-1.5% (m/v) and silicon dioxide were uniformly stirred and mixed in the mass ratio of 20: 50: 20: 7: 3 by using the high-pressure homogenizer. Then those were pressed into 200 mg/tablet by tablet press. When used in animal experiments, the tablets were dissolved in pure water into suspension. The molecular weight of C_60_ present in the obtained FNAO was analyzed using matrix-assisted laser resolution tandem time of flight mass spectrometry (MALDI-TOF-MS, Autoflex III, Bruker, Germany). The 5 *μ*L FNAO (10 *μ*g/mL) was dropped on the copper mesh. After the solution was completely evaporated, the morphology and particle size of FNAO were observed by transmission electron microscopy (TEM, JEOL, JEM-1011, Japan).

**In Vitro Hydroxyl Radical’s Elimination.**

10 *μ*L of DMPO (100 mM) and 15 *μ*L of H_2_O_2_ (100 *μ*M) were mixed with 15 *μ*L ultrapure water, 15 *μ*L excipients suspension without fullerene, and 15 *μ*L FNAO suspension (5 mg/mL). The mixtures were initially exposed to a 500-W UV lamp for 10 seconds, followed by recording the spectrum of DMPO-OH using an X-band electron spin-resonance spectrometer (ESR, Bruker ELEXSYSE, Germany) in the dark. The experiment was repeated three times and the data were analyzed with Origin 2021.

**Zebrafish Methods and Data Analysis**

**Zebrafish**

Zebrafish *Tg(lyz:DsRed2;coro1a:EGFP*) and *Tg(elavl3:YC2)* transgenic lines (obtained from the National Zebrafish Resource Center) and wild type (AB) were used in this study. All fish were reared and maintained at 28 ± 1℃ with a 14 h light: 10 h dark cycle (light on at ZT0 and light off at ZT14). Adult zebrafish were placed in the mating tank on the previous day and separated by a partition board (the ratio of male to female was 1:1 or 1:2). After entering the photoperiod on the second day, the isolation board was removed and embryos were collected. To avoid the formation of pigments, the larvae were kept in embryo medium supplemented with 0.2 mM 1-phenyl-2-thiourea. Breeding was carried out following standard experimental protocols in compliance with the guidelines of the laboratory animal management committee.

**In Vivo Distribution of FNAO by MALDI-MSI.**

The adult wild type zebrafish (2-3 cm full length) were randomly grouped into 4 L fish tanks for the control group and the FNAO-exposed group with 10 fish in each tank. The concentration of FNAO for zebrafish was 25 mg/L/day. Zebrafish were sampled after the 5 days for matrix-assisted laser desorption ionization mass spectrometry imaging (MALDI-MSI) analysis. The zebrafish were extracted and rapidly frozen in liquid nitrogen. Tissue sections were then sliced using a cryo-microtome (Leica CM1950, Nussloch, Germany) set to -20°C. Each zebrafish sample's tissue pieces (40 *μ*m thick) were freeze-thawed and mounted onto an ITO glass slide. The analyses were performed using a MALDI-MSI device, with a repetition rate of 1000 Hz, a sample voltage of 3.00 kV, a detector voltage of 1.90 kV, a laser intensity of 10, and a mass range of m/z 200 - 1000 Da. All mass spectra were recorded, and the dataset was processed and normalized using the MS Solution Version 1.30 program (Shimadzu Corporation, Tokyo, Japan). Regions of interest (ROI) were manually identified.

**Sleep Deprivation (SD) and Treatment**

SD was performed on 6 days post fertilization (dpf) zebrafish larvae during the subjective night for 5 days by maintaining daytime levels of illumination (160 lux). We set four groups including control (Control), sleep deprivation (SD), melatonin (SDM, 1 *μ*M) and FNAO (SDF, 2 *μ*g/mL). For the FNAO treatment group, FNAO was diluted with zebrafish larvae culture medium to prepare a 2 *μ*g/mL FNAO suspension. Replace the newly prepared FNAO suspension every morning of SD, and the FNAO material will enter the digestive tract and intestine of zebrafish as zebrafish breathe and eat. For the melatonin treatment group, 1 *μ*M melatonin solution was prepared by diluting melatonin with zebrafish larvae culture solution. Replace the newly prepared melatonin solution every morning during SD. The control group and SD group were added with the same volume of zebrafish culture solution. All groups were carried out in a 9-cm petri dish, with 30 zebrafish larvae in each group and the culture solution was refreshed daily.

**Locomotor Activity and Sleep Monitoring**

Locomotor activity and sleep were tracked using DanioVision (Noldus). At the end of SD, zebrafish larvae in different groups were transferred into 48-well plates, each containing one larva and 1 mL of embryo medium. The 48-well plate is placed in a chamber filled with pure water and set at a constant temperature of 28℃. Following a brief acclimatization period, swimming behavior was observed for a 24-hour duration, with the initial 14 hours in the light and the final 10 hours in the dark, using a Noldus DanioVision system. Data from the monitors were then analyzed using a custom EthoVision XT 15 software (https://www.noldus.com.cn/daniovision/). The software automatically gives the movement distance and speed of zebrafish, and sets the activity threshold range: 5.00%-50.00%. We define sleep in larval zebrafish as a continuous period of inactivity that lasts at least 1 min [2]. That is if the movement time of zebrafish is less than 0.1 seconds within 1 minute, it is defined as the sleep time of 1 minute. Total sleep time is the sum of ZT0 to ZT14 sleep time and ZT14 to ZT24 sleep time. According to the zebrafish laboratory zeitgeber time (ZT), the circadian rhythm of zebrafish can be accurately studied, and ZT0 is the time point of light (8 a.m.); ZT14 is the time point of dark (10 p.m.).

**FNAO Repair Brain Inflammation**

Zebrafish embryos of *Tg(elavl3:YC2)* transgenic lines were collected according to the required mating method. SD was performed on 6 dpf of zebrafish larvae during the subjective night for 5 days by maintaining daytime levels of illumination (160 lux). We set three groups including control (Control), sleep deprivation (SD), and FNAO (SDF, 2 *μ*g/mL). Zebrafish larvae were anesthetized at the end of SD (zebrafish were immersed in 0.016% Tricaine anesthetic), fixed with 1% low melting point agarose, and photographed by ZEISS stereo fluorescence microscope (AXIO Zoom. V16) as soon as possible.

**Lifespan Assay**

Six days after birth, wild type (AB) zebrafish larvae were randomly divided into three groups: control (Control), sleep deprivation (SD), and FNAO (SDF, 2 *μ*g/mL), 40 in each group. SD was carried out according to the described experimental methods. From the first day of SD, the number of deaths of zebrafish was recorded, and the fresh culture medium was replaced at ZT0 every day, the drug was added and the feed was fed. The survival curve was recorded for 10 days, and the Kaplan-Meier method was used for survival analysis.

**Oxidative Stress Analysis**

The Nanjing Jiancheng Bioengineering Institute (Nanjing, China) provided test kits for measuring the enzyme activity or other biological indicators. To determine how much oxidative stress zebrafish larvae were under, the manufacturer's method was used to measure the levels of malondialdehyde (MDA, A003-1-2), catalase (CAT, A007-1-1), and superoxide dismutase (SOD, A001-3-2). The absorbance was quantified using a SpectraMax iD3 multi-mode microplate reader (USA) and normalized to the total protein levels. 30 zebrafish larvae in each group, with three independent experiments.

**ROS Imaging**

According to our previous report [3], the ROS kit (Nanjing Jiancheng Bioengineering Institute, E004-1-1) was employed to measure ROS levels, following the manufacturer's instructions. DCFH-DA (excitation wavelength: 488 nm; emission wavelength: 525 nm) was added until the final concentration was 10 *μ*M, and then it was left to incubate for 30 minutes in the dark at a constant temperature of 28°C. After that, the zebrafish larvae were washed with fish solution three times and immersed in 0.016% Tricaine anesthetic. A fluorescence microscope (ZEISS, AXIO Zoom. V16) was used to observe and record the images. We used ZEISS software to measure the fluorescence intensity of the ROS staining data. 10 zebrafish larvae in each group, with three independent experiments.

**Migration of Intestinal Immune Cells**

Zebrafish embryos of *Tg(lyz:DsRed2;coro1a:EGFP*) transgenic lines were collected according to the required mating method. The EGFP marked macrophages and the DsRed marked neutrophils in *Tg(lyz:DsRed2;coro1a:EGFP*) transgenic lines. We set four groups including control (Control), sleep deprivation (SD), melatonin (MLT,1 *μ*M) and FNAO (SDF, 2 *μ*g/mL). After 5 days of SD, anesthetized all groups of larval zebrafish (zebrafish were immersed in 0.016% Tricaine anesthetic), fixed with 1% low melting point agarose, and photographed by ZEISS stereo fluorescence microscope (AXIO Zoom. V16) as soon as possible. 10 zebrafish larvae in each group, with three independent experiments.

**Hematoxylin and Eosin (HE) Staining**

Several zebrafish larvae following a 5-day period of SD were collected from the different groups and washed thrice with PBS for 5 minutes and kept in overnight incubation with 4% paraformaldehyde solution (PFA) at 4°C. After dehydration with an ethanol gradient, the fixed larvae were embedded in paraffin wax and sliced into 5 *μ*m sections using a Leica microtome. The H&E staining procedure was carried out following previously published protocols [3], and images were observed and photographed with a microscope (Leica DM2500, Germany). 5 zebrafish larvae in each group, with three independent experiments.

**Mice Methods and Data Analysis**

**Mice**

Male C57BL/6 mice (7 weeks old) were purchased from Beijing Huafukang Bioscience Co. Inc. (Beijing, China). All mice were housed in a standardized animal room with regulated temperature and ventilation, and provided ad libitum access to food and water. The mice were maintained on a 12-hour light/dark cycle, with lights on at ZT0 and lights off at ZT12. In addition, all mice were given sterile water and a standard normal chow diet which were procured from Beijing Huafukang Bioscience Co. Inc. (Beijing, China). All animal experiments were conducted in accordance with the National Regulation of China for Care and Use of Laboratory Animals. The experimental protocols involving live animals were reviewed and approved by the Animal Ethics Committee of the Institute of Chemistry, CAS.

**SD and Treatment**

To induce continuous SD, the mice were placed in the Mouse Sleep Deprivation Apparatus (KW-BD, KEWBASIS), which featured a rotating bar positioned at a short distance above the cage floor. The bar was kept in constant, gentle motion (speed 14) to restrict sleep. Age-matched control mice that were not sleep-deprived were placed in a similar-sized apparatus without the rotating bar. We set three groups including control (Control), sleep deprivation (SD), and FNAO (SDF, 5 mg/mL). During SD, mice in the SDF group were treated with FNAO (40 mg/kg, intragastric administration, i.g.) at ZT2 and ZT11 each day, and the SD group mice were fed the same concentration of excipients as control. 8 mice in each group, with three independent experiments.

**Sleep Recording Electrode Placement, Sleep Monitoring and Scoring**

After anesthesia by gas anesthesia machine (3% isoflurane anesthesia was induced to maintain 1.5%), the mice were fixed on a stereotaxic apparatus, and the scalp was shaved and disinfected. Cut the fascia of the top skin of the craniotomy, smear the surface of the skull with a cotton ball dipped in a small amount of hydrogen peroxide to expose the Bregma point clearly, drill the skull through the corresponding frontal lobe and parietal lobe with electric coagulation scalpel, but pay attention to avoid injuring the brain parenchyma. Place head electrode (EEG) and neck electrode (EMG) (electrode is welded on 2 × 4 Pin channel connector in advance) and fixed with dental glue. Suture the wound after complete solidification of dental gel. Put the mice back in the cage and recover for a week.

After 7 days of SD in mice, EEG signals were continuously collected for 24 hours. The signals were first collected by Powerlab 15T data acquisition system, and then recorded in real-time by Labchart software (version8.1.16). The EDF format was exported and analyzed by SleepSign software. According to the standard, the sleep-wake state is divided into three states: wakefulness (Wake), NREM sleep and REM sleep. The main criterion of the awakening stage is low amplitude, mixed frequency EEG activity (the delta wave ratio in each frame is less than 30%), and relatively high amplitude EMG activity; the main standard of NREM sleep is that slow wave (0.5-4 Hz) is relatively more active (delta wave ratio in each frame is greater than 30%), and the EMG amplitude is relatively low. The main standard of REM sleep is the fast frequency of theta wave (5-10 Hz) dominated EEG activity and EMG activity continues to be the lowest state. According to the mice laboratory zeitgeber time (ZT), the circadian rhythm of mice can be accurately studied, and ZT0 is the time point of light (7 a.m.); ZT12 is the time point of dark (7 p.m.).

**Immunohistochemistry**

Mice were deeply anesthetized by a gas anesthesia machine (3% isoflurane anesthesia was induced to maintain 1.5%) and perfused transcardially with 0.9% normal saline and 4% paraformaldehyde. Brains were post-fixed in 4% paraformaldehyde at 4℃ for 48 hours. For Nissl staining, the fixed brains were embedded in paraffin wax and cut into 5 *μ*m slices with a Leica microtome. Afterward, the paraffin sections were deparaffinized, cleaned, stained (Toluidine Blue O Solution, 1%, Solarbio, G3668), cleaned and sealed successively.

For Iba-1 immunohistochemistry staining, 5-*μ*m-thick paraffin sections were incubated overnight with a primary antibody (Anti-Iba-1, abcam, ab178847) at 4℃. Anti-beta amyloid (Rabbit polyclonal to beta amyloid, ab2539, Abcam, 1:100) was used and was detected with biotinylated secondary antibody for 1 h in the next day.

All Nissl staining and Iba-1 immunohistochemistry staining sections were imaged using the digital pathology scanner (Nano Zoomer-SQ, Japan) and photo images were analyzed with the Image-Pro Plus 6.0 software.

For ZO-1 immunofluorescence staining, the brains, which had been cryo-protected, were sliced into 40-micron sections using a cryostat microtome (Leica). The brain sections were then washed five times for 5 minutes each in PBST (0.3% Triton X-100 in PBS) and subsequently incubated in a blocking solution (5% Normal Donkey Serum, Solarbio, SL050) at room temperature for 1 hour. The brain sections were then exposed to primary antibodies and incubated overnight at 4℃. Subsequently, immunofluorescence-tagged secondary antibodies (Beyotime, A0453) were applied to the sections and incubated at room temperature for 2 hours. The nuclei were stained with DAPI (Beyotime, P0131). Thereafter, the images of brain sections were observed with the confocal microscopy (Olympus).

**ELISA**

The intestinal tissues (the ileum tissue or the colon tissue), 1 cm from each mouse, was homogenated in 500 *μ*L RIPA (Solarbio) on the ice. The tissue slurry was centrifuged at 12000 rpm for 30 min at 4°C. The blood of the mice was collected from the orbital sinus. The blood was centrifuged at 3000 rpm for 10 min at 4°C. Moreover, the supernatant and serum were collected for evaluation. Commercial mouse IL-1*β*, IL-6, and TNF-*α* *ELISA* kit (Jianglai, China) and mouse 5-HT, Melatonin *ELISA* kit (Elabscience) were used to quantify the homogenate of intestinal tissues and the serum of mice.

**ROS Measurement**

After 7 days of SD, the mice were sacrificed, and the intestinal contents were washed with 0.9% normal saline. We evaluated intestinal ROS levels in SD mice by two methods.

The intestinal cells were digested with collagenase to obtain intestinal cells. After washing with dPBS, the intestinal cells were incubated at 37°C for 30 minutes with a commercial ROS detection kit (S0033 M). The intestinal cells were washed twice with 1 × PBS and filtered into the flow tube. The flow cytometry (BD, USA) was used to detect the excitation wavelength of 488 nm. 2 × 10^4^ cells were collected from each sample. FlowJo VX software was used to analyze the results and make quantitative statist

The ileum tissue and the colon tissue were collected and embedded in OCT, then these samples were frozen immediately at -20°C and sliced into pieces on a Leica cryostat within in 6 hours post-dissection. Next, 30 *μ*m sections were stained with dihydroethidium (DHE) (Beyotime Biotechnology) according to the manufacturer’s instruction for 30 minutes at 37°C. The nuclei were stained with 6-diamidino-2-phenylindole (DAPI) (Beyotime Biotechnology). Thereafter, the images of these slices were observed and obtained with the confocal microscopy (Olympus).

**Western Blot**

The protocol for western blot (WB) was based on our previous studies [4]. WB was performed to quantify the protein expression of ZO-1 (Abcam), Occludin (Abcam), NF-*κ*B p65, P-NF-*κ*B p65, IKK*β*, P-IKK*β*, I*κ*B-*α* and P-I*κ*B-*α* (CST) in the intestinal tissue. *β*-actin (CST) was performed as an internal reference protein.

**Statistical Analysis.**

Data are presented as mean ± SD unless indicated otherwise. Independent - Sample *t*-test was used to compare data between two independent groups. One-way analysis of variance (ANOVA) followed by an LSD test was performed to analyze the difference between multiple groups. All data were analyzed using IBM SPSS Statistics 27 (IBM, USA). The figure legends provide information on the number of biological replicates and statistical methods used. Differences in the figures are indicated by *P* values or asterisks. * represents *p* < 0.05, ** represents *p* < 0.01, *** represents *p* < 0.001 compared with the model group. # represents *p* < 0.05, ## represents *p* < 0.01, and ### represents *p* < 0.001 compared with the control group.


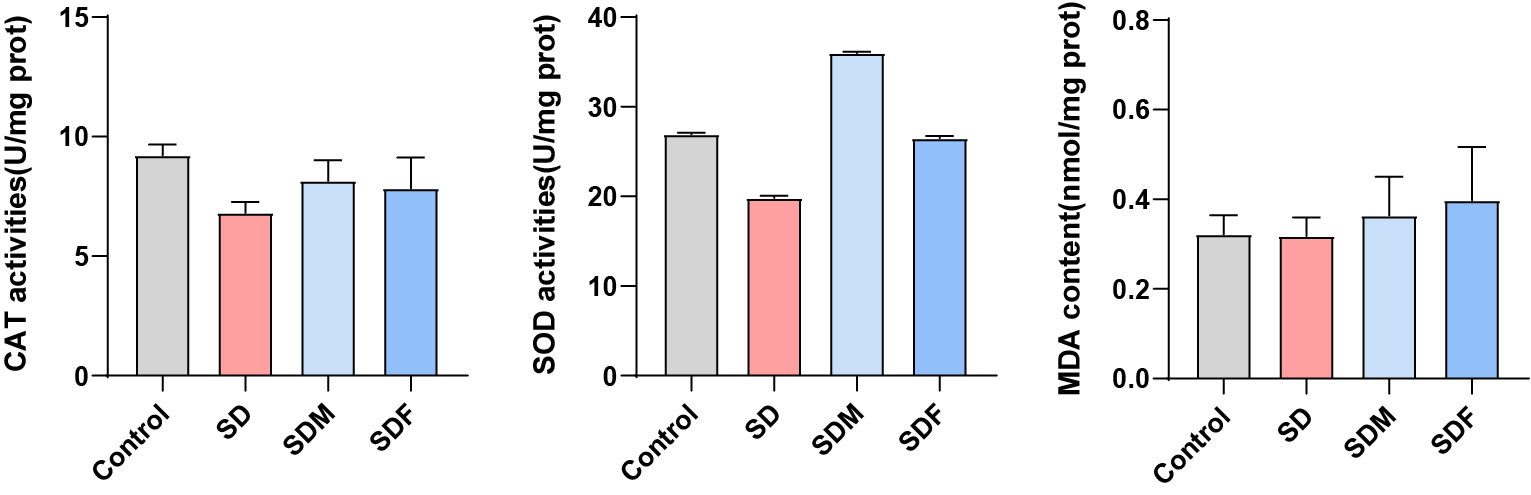


**Figure S1.** Measurements the level of CAT activities, SOD activities, and MDA content of the zebrafish after 1 day of SD.

**
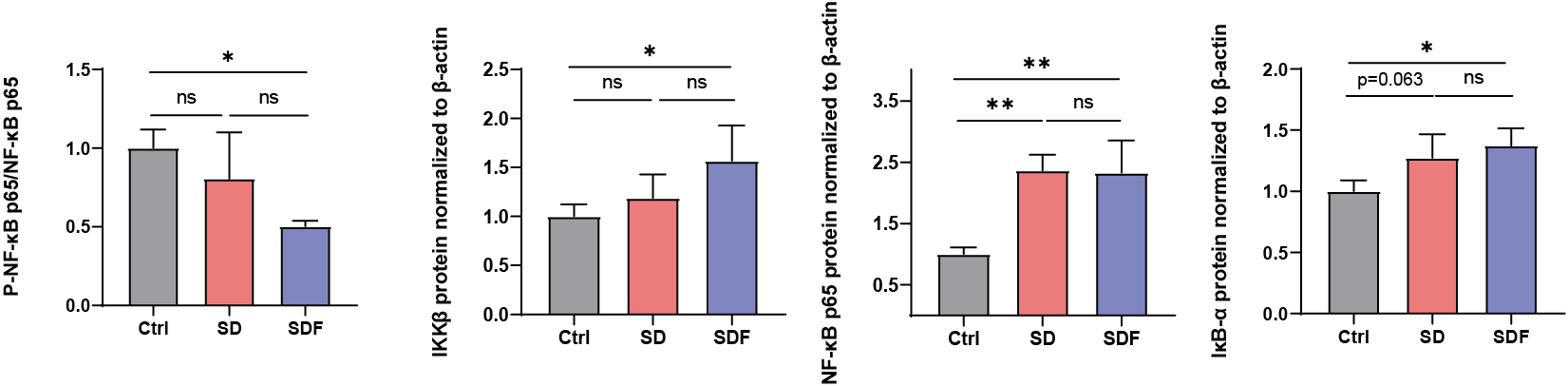
**

**Figure S2.** The quantification of expression of other proteins in the NF-*κ*B signaling pathway.


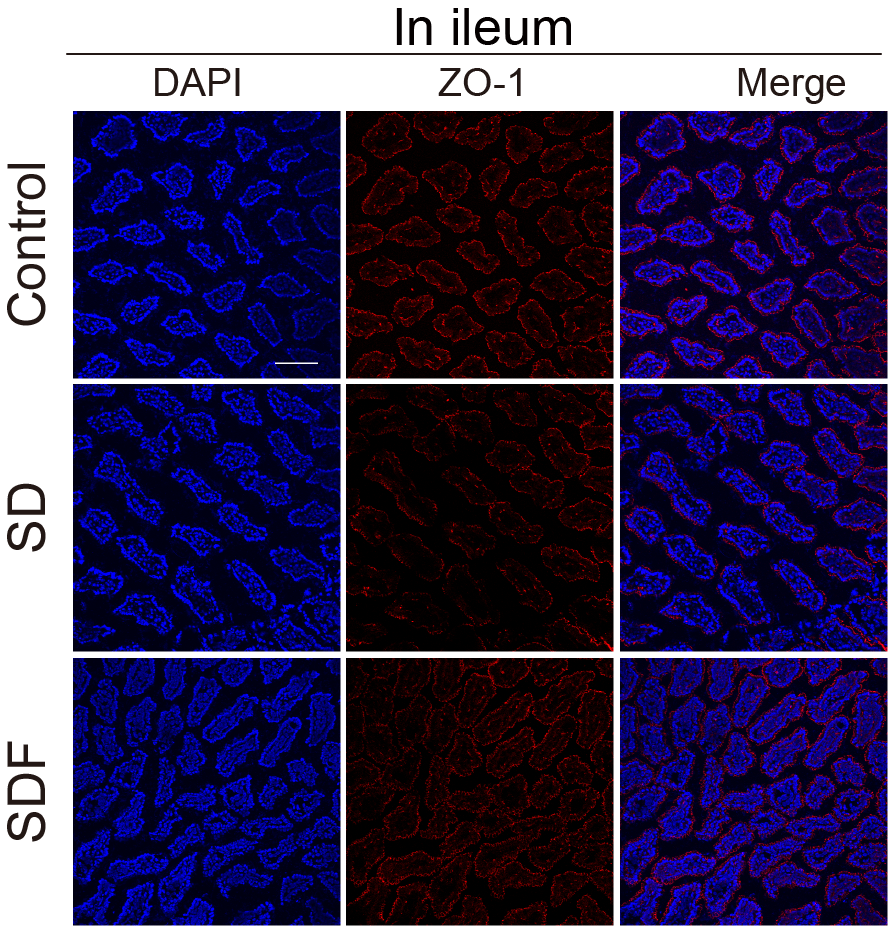


**Figure S3.** ZO-1 immunofluorescence images in the ileum of the mice, scale bar is 100 *μ*m.


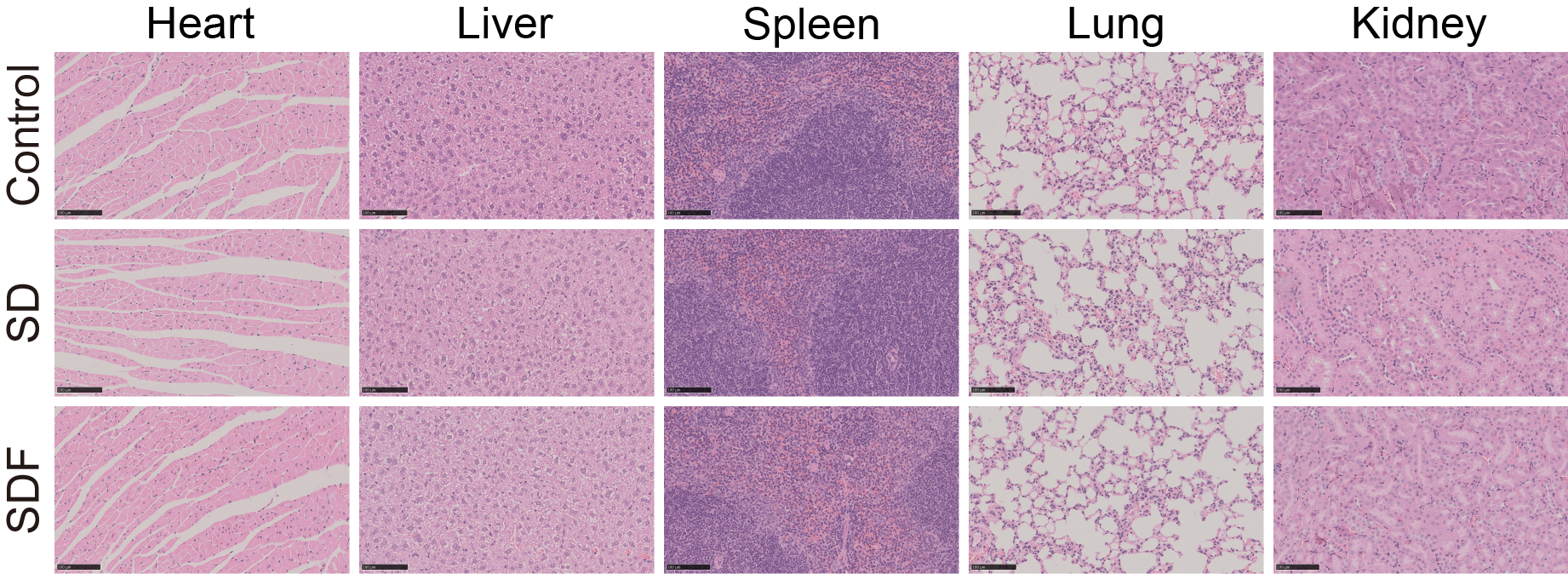


**Figure S4.** H&E staining images after FNAO treatment. Scale bars, 100 *μ*m.

References

1. Cao XR, Zhen MM, Li L *et al.* Oral fullerene tablets for colorectal cancer therapy based on modulation of tumor inflammatory microenvironments. *J. Mater. Chem. B.* 2022; **10**: 9457-65.

2. Rihel J, Prober DA, Arvanites A *et al.* Zebrafish Behavioral Profiling Links Drugs to Biological Targets and Rest/Wake Regulation. *Science* 2010; **327**: 348-51.

3. Fan GQ, Shen TZ, Jia K *et al.* Pentachloronitrobenzene Reduces the Proliferative Capacity of Zebrafish Embryonic Cardiomyocytes via Oxidative Stress. *Toxics* 2022; **10**: 17.

4. Jia W, Zhen M, Li L *et al.* Gadofullerene nanoparticles for robust treatment of aplastic anemia induced by chemotherapy drugs. *Theranostics* 2020; **10**: 6886-97.
